# Neonatal Hypoxia, Hippocampal Atrophy, and Memory Impairment: Evidence of a Causal Sequence

**DOI:** 10.1093/cercor/bht332

**Published:** 2013-12-15

**Authors:** Janine M. Cooper, David G. Gadian, Sebastian Jentschke, Allan Goldman, Monica Munoz, Georgia Pitts, Tina Banks, W. Kling Chong, Aparna Hoskote, John Deanfield, Torsten Baldeweg, Michelle de Haan, Mortimer Mishkin, Faraneh Vargha-Khadem

**Affiliations:** 1 Developmental Cognitive Neuroscience Unit; 2 Imaging and Biophysics Unit; 3 Cardiac Unit,UCL Institute of Child Health, London WC1N 1EH, UK; 4 UCL Institute of Cardiovascular Science,London WC1E 6BT, UK; 5 Cardiac Intensive Care; 6 Department of Radiology; 7 Department of Neuropsychology, Great Ormond Street Hospital, London WC1N 3JH, UK; 8 Laboratory of Neuropsychology, National Institute of Mental Health, Bethesda, MD 20892, USA; 9 Murdoch Childrens Research Institute, Melbourne, Victoria 3052, Australia; 10 University of Castilla-La Mancha, Albacete 02006, Spain; 11 Freie Universität, Berlin 14195, Germany

**Keywords:** developmental amnesia, hippocampal volumes, hypoxia

## Abstract

Neonates treated for acute respiratory failure experience episodes of hypoxia. The hippocampus, a structure essential for memory, is particularly vulnerable to such insults. Hence, some neonates undergoing treatment for acute respiratory failure might sustain bilateral hippocampal pathology early in life and memory problems later in childhood. We investigated this possibility in a cohort of 40 children who had been treated neonatally for acute respiratory failure but were free of overt neurological impairment. The cohort had mean hippocampal volumes (HVs) significantly below normal control values, memory scores significantly below the standard population means, and memory quotients significantly below those predicted by their full scale IQs. Brain white matter volume also fell below the volume of the controls, but brain gray matter volumes and scores on nonmnemonic neuropsychological tests were within the normal range. Stepwise linear regression models revealed that the cohort's HVs were predictive of degree of memory impairment, and gestational age at treatment was predictive of HVs: the younger the age, the greater the atrophy. We conclude that many neonates treated for acute respiratory failure sustain significant hippocampal atrophy as a result of the associated hypoxia and, consequently, show deficient memory later in life.

## Introduction

In several earlier reports (Vargha-Khadem et al. 1997; Gadian et al. 2000; de Haan et al. 2006a; Adlam et al. 2009), we described a series of children and adolescents who had come to medical attention because of severe long-term memory impairment, which we subsequently found was due to selective bilateral hippocampal atrophy. This disorder was labeled developmental amnesia (DA) (Gadian et al. 2000), a form of amnesia characterized by impairment in episodic or event memory (Vargha-Khadem et al. 1997), with relative sparing of semantic or fact memory. The mnemonic selectivity of the disorder is most likely attributable to mediation of semantic memory by structures, including in particular the parahippocampal region (Mishkin et al. 1997; Vargha-Khadem et al. 1997), that are either unaffected or much less affected than the hippocampus (Gadian et al. 2000).

Examination of medical records in each DA case revealed a history of hypoxia-ischemia suffered perinatally or later in childhood due to cardiac, respiratory, or other neurological disorders or their treatments. Typically, the memory problems gradually unfolded during later childhood, long after the initial exposure to hypoxia-ischemia, suggesting that, in normal individuals, in addition to anatomical maturation of the hippocampus, there is also functional maturation of hippocampus-dependent memory processes (Bachevalier and Vargha-Khadem 2005).

It is well established that brain structures in neonates, like those in adults, differ in their vulnerability to hypoxia-ischemia, with the basal ganglia, thalamus, and hippocampus showing particular susceptibility to such early-life episodes both in animals (Raman et al. 2006; Golan et al. 2009; Rodricks et al. 2010) and in humans (Van Paesschen et al. 1997; Mañeru et al. 2003; Rutherford et al. 2004; Okereafor et al. 2008).

Hypoxia-induced cell loss in the neonatal basal ganglia and thalami can lead to the motor problems of cerebral palsy and, in humans, speech problems (Okereafor et al. 2008) manifesting early in life as oromotor dyspraxia. In contrast to, and even in the absence of, these motor effects of injury to the basal ganglia and thalamus, damage to the neonatal hippocampus can initially have no overt cognitive or behavioral consequences, but manifests later in memory difficulties both in animals (Dikova et al. 1993; Titus et al. 2007; Huang et al. 2009; Rodricks et al. 2010) and in children (Gadian et al. 2000; Isaacs et al. 2003; Mañeru et al. 2003; Vargha-Khadem et al. 2003; de Haan, Mishkin et al. 2006a; de Haan, Wyatt et al. 2006b; Raman et al. 2006).

The foregoing narrative of how a hypoxic-ischemic episode sustained early in life could lead initially to hippocampal atrophy, and how this atrophy could lead later in childhood to severe memory problems seems well supported by the evidence. In fact, however, the entire sequence was uncovered in reverse order: memory problems were observed first, hippocampal atrophy was found next, and hypoxia-ischemia sustained early in life was uncovered last. Because the explanation was thus arrived at post hoc, we cannot be certain that the hypoxic-ischemic episode was indeed the causal event. A far stronger case for the causal sequence could be made if a cohort of children were selected on the basis of the presumed initial causal event and were then found to have the consequent neural and behavioral effects. The present experiment was designed to test this predicted sequence.

The cohort selected were children who, as neonates, had acute hypoxemic respiratory failure (AHRF) and were thus potentially at risk of sustaining hippocampal atrophy and, consequently, of suffering from some degree of memory impairment later in life. It should be noted that hypoxemia (i.e., low blood oxygen) is not synonymous with tissue hypoxia, but whereas hypoxemia can be measured via biochemical methods, tissue hypoxia (defined as a decrease in the partial pressure of oxygen at the cellular level) cannot be directly and noninvasively measured in critically ill patients within the intensive care environment. However, direct measures assessing the effect of full cessation of circulation (hypothermic circulatory arrest) in animal surgical studies clearly demonstrate the relationship between induced hypoxemia and low global oxygenation (circulatory arrest or selective cerebral perfusion) with low brain tissue oxygenation and subsequent histopathological injury (Pokela et al. 2001; Salazar et al. 2009). These neonates were treated either by conventional intensive care management (CM) or, in cases unresponsive to standard intensive care measures, by an advanced life support system (i.e., extracorporeal membrane oxygenation, ECMO) supporting the heart and lungs to allow recovery of cardiopulmonary function. Results of a previous study (McNally et al. 2006) suggest that memory function may indeed be impaired in children with this history of oxygen deprivation and treatment. To address the issue more fully, we undertook a thorough neuroimaging and neuropsychological assessment of our selected cohort. Our main aim was to determine whether any of the children in the cohort had DA and, if so, to obtain an estimate of the incidence of this disorder.

## Materials and Methods

### Participants

Based on the records held by the National Perinatal Epidemiology Unit (NPEU), University of Oxford, and by Great Ormond Street Hospital (GOSH), 159 children who had received treatment for AHRF as neonates at 1 of 5 centers within the UK (Great Ormond Street Hospital, London; Glenfield Hospital, Leicester; the Freeman Hospital, Newcastle; the Royal Hospital for Sick Children, Glasgow; and Kings College Hospital, London) were eligible to participate in this study. These 159 children were sent letters, either from the NPEU or from GOSH, inviting them to participate in the study. A total of 60 invitees responded to the letters and consented to participate in the study, but 20 patients who consented (33%) were excluded because they either failed to attend the neuropsychological testing sessions (*N* = 11), refused or were unable to undergo brain scanning (*N* = 4), had full scale IQs below 75 (*N* = 2), had special educational needs (*N* = 2), or had highly discordant scores on the Children's Memory Scale and the Rivermead Test of Everyday Memory (*N* = 1). Thus, the final cohort of 40 children (i.e., 67% of those who consented; *M*_age,_ 12:2 [years:months] ± 1:8 SD; 24 males) met the following inclusionary criteria: 1) 8–15 years of age at the start of the study; 2) no overt neurological impairment or diagnosis of motor, learning, or cognitive difficulties; 3) free of genetic syndromes; and 4) native English speakers residing in the UK. This cohort underwent a structural MRI scan and a detailed neuropsychological evaluation spanning an entire day. The low rate of response (60 of 159) to our recruitment effort could have been due to a number of factors, such as: 1) long distance travel, 2) time off work for parents accompanying their children for visits to GOSH, 3) “research fatigue” in children and their families who had been recruited into the original ECMO clinical trial (UK Collaborative ECMO Trial Group 1996), as this included 3 follow-up studies at 1, 4, and 7 years (UK Collaborative ECMO Group 1998; Bennett et al 2001; McNally et al 2006, respectively) to determine long-term morbidity after ECMO versus CM for treatment of AHRF.

Of the 40 children comprising the full cohort, 27 (68%) had received treatment with ECMO, and 13 (32%) had received CM. Seventeen cases receiving ECMO, and 11 receiving CM were part of a randomized clinical trial to evaluate long-term outcome after ECMO treatment in terms of mortality and morbidity (UK Collaborative ECMO Group 1998; McNally et al. 2006). For the cohort of babies in the ECMO study, there were almost twice the number of survivors in the ECMO group as there were in the conventional management group. As a result, there were twice the number of babies potentially available for follow-up (ECMO = 56, CM = 34 at 7-year follow-up of the cohort that had participated in the randomized clinical trial). A similar disparity was also observed in those children who were recruited from GOSH. For babies not in the ECMO trial, most referrals to GOSH receive ECMO when conventional treatment has failed. While about one-third of these babies continue to improve with ongoing conventional therapy at GOSH, most (i.e., ∼ 2/3) go on to receive ECMO as a proven rescue therapy. In the majority of cases comprising the entire cohort, the most frequent cause of the AHRF was meconium aspiration syndrome (see Supplementary Table 1 for further details). Gestational age in the patient cohort ranged from 34 to 42.6 weeks (although only 3 patients had a gestational age below 37 weeks—one each at 34, 35, and 36.5 weeks; removal of these 3 patients did not change either the behavioral or neuropathological results; see below). Values of medical variables made available by the NPEU in the initial assessment are presented in Table 1 (see Supplementary Table 2 for comparison of medical variables in the ECMO and CM subgroups of both the full and randomized trial cohorts).

**Table 1:** Medical variables noted at initial assessment during neonatal period of the acute hypoxemic respiratory failure (AHRF) cohort

Medical variables	*N*	Mean	SD	Median	IQR
Current age (years:months)	40	12.2	1.8	12.5	11.0–13.5
Gestational age (weeks)	39	39.5	2.1	40.0	37.8–41.0
Birth weight (kg)	39	3.5	0.7	3.5	3.0–3.8
Arterial pH	37	7.3	0.1	7.3	7.2–7.4
Arterial CO_2_ level (kPa)	38	5.9	2.0	5.9	4.5–7.1
Arterial PaPO_2_ level (kPa)	38	5.1	1.6	5.0	3.8–6.2
Oxygenation index	38	61.8	21.9	52.2	45.8–81.1
Apgar (5 mins)	12	7.1	2.2	7.0	5.0–9.5

CO_2_, carbon dioxide level in arterial blood; IQR, interquartile range; kg, kilograms; kPa, kilopascals; mins, minutes; PaPO_2_, partial pressure of oxygen; pH, the acidity/alkalinity of arterial blood; SD, standard deviation.

The patients' socioeconomic status, as judged by parental occupations (Ganzeboom et al. 1992), and their behavioral and social problems, as judged by parental ratings (Achenbach 1991), were within the normal range (Table 2). Comparison of parental occupations and behavior and social profiles in the ECMO and CM subgroups of both the full and randomized trial cohorts did not reveal any significant subgroup differences (see Supplementary Tables 3 and 4, respectively).

**Table 2:** Socioeconomic Status and Child Behavior Checklist ratings of the AHRF cohort

A. Socioeconomic Status^a^	*N*	Mean value	SD	Range
Father's occupation	34	49.4	21.4	23–88
Mother's occupation	29	49.4	17.9	23–88
B. Child Behavior Checklist (CBC)^b^	*N*	Mean (*T*-score)	SD	Range
Anxious-depressed	36	54.2	7.1	50–79
Withdrawn-depressed	36	54.8	6.0	50–73
Somatic complaints	36	56.3	7.1	50–78
Social problems	36	55.3	7.4	50–70
Thought problems	36	55.7	7.3	50–73
Attention problems	36	57.9	9.4	50–83
Delinquent behavior (Rule Breaking)	36	52.5	4.3	50–71
Aggressive behavior	36	53.4	6.2	50–75

Note: ^a^Parental occupation classified according to the International Standard Classification of Occupation (ISCO-88) transformed into an International Socioeconomic Index of Occupational Status (Ganzeboom et al. 1992) to provide socioeconomic values for the parental occupations with a range of 0–90.

^b^The Child Behavior Checklist (CBC; Achenbach 1991), designed to assess behavioral and social problems of children and adolescents as judged by parental ratings, uses *t*-scores with a mean of 50 (SD 10) where <60 = normal range of functioning; 60–70 = borderline range; and >70 = clinical range. SD, standard deviation.

Sixty-four healthy children recruited from Greater London mainstream schools or from the pool of healthy siblings of patients attending Great Ormond Street Hospital (NC group: *M*_age_, 11:5 years: months ± 2:1 SD; 28 males) provided control data for the MRI scans as well as norms for behavioral tests lacking standardized means.

The study was approved by the Research Ethics Committee of University College London Hospital, and both the children and their parents gave informed consent.

### Imaging Procedures

Whole-brain MRI scans were obtained using a 1.5-T Siemens Avanto scanner, with a *T*_1_-weighted 3D FLASH sequence: repetition time 11 ms, echo time 4.94 ms, flip angle 15°; matrix size, 224 × 256; field of view, 250 mm; partition thickness, 1 mm; 176 sagittal partitions in the third dimension; acquisition time, 5.34 min.

For the measurement of hippocampal volumes (HVs), the datasets were reformatted into 1-mm-thick contiguous slices in a tilted coronal plane perpendicular to the long axis of the hippocampus using MEDx 3.43 (Medical Numerics, Inc., MD, USA). Hippocampal boundaries were selected on the basis of a survey of numerous anatomical studies and brain atlases and were adapted to enable reproducible ROI tracing within and across brains (Jack et al. 1995; Duvernoy 1988; Duvernoy and Bourgouin 1998, 1999; Insausti et al. 1998; Bernasconi et al. 2000; Goncharova et al. 2001; Duvernoy 2005).

The hippocampus was defined as a composite of the following regions: cornu Ammonis subfields (CA1-4), dentate gyrus, subiculum and presubiculum, amygdalo-hippocampal transition area, and uncus. The rostral boundary, marking the division between hippocampus and amygdala, was set at the alveus and anterior tip of the temporal horn of the lateral ventricle, with the ventricle serving also as the lateral boundary. The medial boundary, marking the division between hippocampus and entorhinal cortex, was placed at the dorsomedial edge of the temporal lobe, except at the rostral and caudal tips of the hippocampus. The caudal boundary was set to include the last slice in which the hippocampus could be distinguished from the fornix.

Hippocampal cross-sectional areas were measured by one of the authors (D.G.G.) along the length of the hippocampus, using every slice (see also, Maller et al. 2006). The volumes were calculated by summing the cross-sectional areas and multiplying by the distance between the measured slices. All measurements were carried out blind to the clinical data.

Total cortical gray matter (GM), white matter (WM), and cerebrospinal fluid (CSF) volumes were obtained from the new segmentation procedure implemented in SPM8 (http://www.fil.ion.ucl.ac.uk/spm/software/spm8/). A correction was made for all the measured regions (GM, WM, CSF, and HV) using a linear regression that removed the contribution of intracranial volume (ICV). Regression coefficients were derived from participants in the control group, but the correction was applied to all participants. All volume measures are presented here in this corrected form.

Measurements were made manually in preference to automated methods, because any automated method would have required validation for this patient population. Moreover, in terms of accuracy and precision, manual tracing is still the gold standard for measuring regional volumes (Chupin et al. 2007; Klein et al. 2009). Indeed, the absence of clear boundaries in MRI scans between the hippocampus and the amygdala renders the segmentation of these structures in MNI space a challenge for most automatic registration methods (Chupin et al. 2009).

### Neuropsychology

Participants were assessed for overall intellectual ability with the Wechsler Intelligence Scale for Children–4th Ed (WISC-IV) (Wechsler 2003), for academic attainments with the Wechsler Individual Attainment Test–2nd Ed. (WIAT-II) (Wechsler 2001), and for verbal fluency and word generation with the subtests of the Delis–Kaplan Executive Function System (D-KEFS) (Delis et al. 2001).

The Children's Memory Scale (Cohen 1997) provided measures of immediate and delayed visual and verbal memory yielding a combined memory quotient (MQ), as well as measures of learning, delayed recognition, and attention/concentration. For each patient, a predicted MQ was obtained from that patient's full scale IQ and compared with the actual MQ (Drozdick et al. 2008) to provide an additional measure of memory impairment. For each patient, the discrepancy (and its significance level) between the IQ-predicted MQ and the Actual MQ was obtained from the CMS manual based on values of the WISC-III full-scale IQ. The CMS tables indicate that the WISC-III and WISC-IV MQ predictions at each IQ level (Drozdick et al. 2008) are highly consistent. However, there are currently no significance levels associated with WISC-IV MQ predicted scores. Therefore, patient scores were checked for significance levels using predictions from the WISC-III. Memory for everyday events was evaluated with the Rivermead Behavioral Memory Test (RBMT) (Wilson et al. 1985) for participants aged 11–15 years and with the children's version (Wilson et al. 1991) for those aged 8–10:11 years.

### Statistical Methods

*t*-Tests were used to compare the ICV-corrected brain-region volumes of the patient cohort with those of the normal control group and to compare the cohort's mean scores on the standardized neuropsychological tests with the standard population means. Nonparametric Mann–Whitney *U*-tests were used to compare the cohort and control groups' mean profile scores on the nonstandardized RBMT.

Some of the neuropsychological tests, particularly those assessing memory, intelligence, and academic achievement, were highly correlated. To determine the extent to which these cognitive domains were represented independently of each other in this cohort, and to counter the risk of false-positive errors resulting from multiple comparisons, we carried out a principal component analysis (PCA) on the standardized scores of WISC, WIAT, and CMS indices, using eigenvalues >1 as a cutoff criterion and Varimax rotation. Stepwise multiple linear regression and Pearson's correlation analyses were performed to examine the relationships 1) between brain-region volumes and memory measures (MQ, mean Visual/Verbal Immediate Memory and, separately, Delayed Memory), 2) between medical variables (Table 1) and HVs (left, right, and bilateral—i.e., the mean of left and right volumes), and 3) between medical variables and memory measures.

## Results

### Brain-Region Volumes of the AHRF Cohort

**Figure 1.**
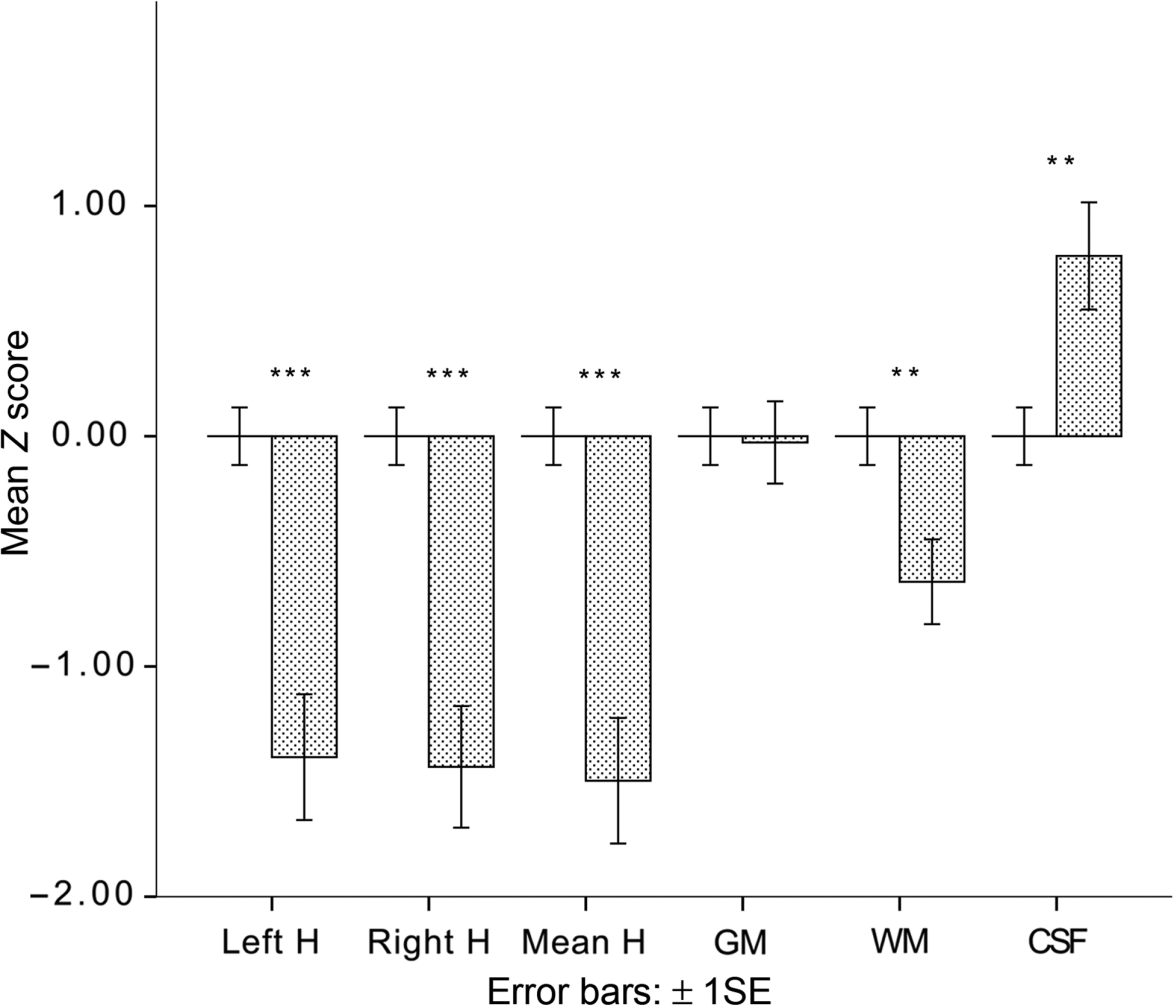
ICV-corrected hippocampal and global brain volumes in 40 AHRF patients against a mean of 64 controls (set at *z* = 0). H, hippocampus; GM, gray matter; WM, white matter; CSF, cerebrospinal fluid. **P < 0.01; ***P < 0.001 (independent samples *t*-test, df adjusted).

Using the mean and standard error of the control group's brain-region volumes as baseline, the cohort's volumes were transformed into *z*-scores to allow direct comparison of the cohort's deviation from baseline across the widely different volumes of the 4 brain regions. As shown in Figure 1, the mean volumes of the cohort's left, right, and bilateral hippocampi were significantly below baseline (all *P*'s **<**0.001). The volumes of 2 other brain regions showed smaller but significant differences (both *P* values = 0.01) between the cohort and control group: one was a reduction in the cohort's WM, and the other, an increase in the cohort's cerebrospinal fluid, the increased CSF presumably reflecting the reduction in WM as well as in HVs.

The degree of hippocampal atrophy in the ECMO and CM subgroups did not differ significantly in either the full or the randomized trial cohort, but an increase in CSF volume in the ECMO relative to the CM subgroup was observed in both cohorts. Although the CSF increase in the ECMO subgroup was not accompanied by significantly decreased WM (as it was in the full cohort), there was a trend in this direction (reduced WM in the ECMO compared to the CM subgroup, *P* = 0.09; see Supplementary Table 5 for further details).

### Cognitive Profile of the AHRF Cohort

The neuropsychological results of the full AHRF cohort are listed in Table 3. Statistical comparisons of the neuropsychological results between the ECMO and CM subgroups of both the full cohort and the randomized trial cohort are shown in Supplementary Table 6.

**Table 3:** Cognitive profile of the AHRF cohort a

Cognitive domain	AHRF cohort (*N* = 40)			
	Mean SS	SD	*t*-test (df = 39)	*P* value
A.				
Intelligence (WISC-IV)				
Full Scale IQ	97	13	−1.30	0.20
Verbal Comprehension	96	12	−1.88	0.07
Perceptual Reasoning	100	14	−0.19	0.85
Working Memory	96	11	−2.05	**0.047**
Processing Speed	99	14	−0.31	0.76
Academic Attainments (WIAT)				
Word Reading	98	12	−1.19	0.24
Reading Comprehension	102	14	0.80	0.43
Spelling	95	12	−2.78	**0.008**
Numerical Operations	96	17	−1.59	0.12
Mathematical Reasoning	97	12	−1.38	0.18
Verbal Fluency (D-KEFS)				
Letter Fluency	99	17	−0.56	0.58
Category Fluency	107	14	3.13	**0.003**
B.				
Memory (CMS)				
General Memory Quotient	87	17	−5.03	**<0.001**
Verbal Immediate	86	15	−5.97	**<0.001**
Verbal Delayed	86	16	−5.79	**<0.001**
Visual Immediate	100	12	−0.17	0.87
Visual Delayed	90	14	−4.76	**<0.001**
Attention/Concentration	102	14	0.78	0.44
Learning	93	13	−3.15	**0.003**
Delayed Recognition	84	19	−5.55	**<0.001**
Mean of Visual/Verbal Immediate	93	12	−3.74	**0.001**
Mean of Visual/Verbal Delayed	88	13	−6.00	**<0.001**
Episodic Memory	Median (AHRF)	Median (Controls)	*U*	*P*
Rivermead Behavioral Memory Test (/22)	17	21	780	**0.001**

Note: ^a^Age at test, 8–15 years. *t*-Tests indicate comparison of standardized scores of the cohort with the standard population mean of 100 ± 15(SD). *U*-value indicates the comparison between the Rivermead Behavioral Memory scores of the cohort and the 64 healthy controls using the nonparametric Mann–Whitney *U*-test. Significant *P* values are shown in bold. Abbreviations: AHRF, acute hypoxemic respiratory failure; CMS, Children's Memory Scale; D-KEFS, Delis-Kaplan Executive Function System; SD, standard deviation; SS, Standardized score; WIAT-II, Wechsler Individual Attainment Test; WISC-IV, Wechsler Intelligence Scale for Children IV.

#### Intelligence

The scores of the patient cohort did not differ significantly from those of the standard population (*M* = 100, SD = 15) either in Full Scale IQ or in 3 of the 4 domains composing it: Verbal Comprehension, Perceptual Reasoning, and Processing Speed. The one exception was in the domain of Working Memory, which showed a small but significant deficit (*P* < 0.05). Examination of the Working Memory scores in the ECMO and CM subgroups indicates that they differed in the randomized trial cohort but not in the full cohort, raising questions as to the reliability of the difference in the 2 subgroups, particularly because in this case it was the CM subgroup that fell significantly below the ECMO subgroup, which was the more consistently impaired of the 2 (see below).

Comparison of the 2 subgroups' scores on Full-Scale IQ and its subdomains indicated a more consistent finding of significantly lower Verbal Comprehension (VC) in the ECMO than in the CM subgroup in both the full cohort (*M*_VC_, 94 vs. 102*, P* = 0.02) and in the randomized trial cohort (*M*_VC_, 92 vs. 101, *P* = 0.05).

#### Academic Attainments

The cohort's mean scores on the literacy and numeracy subtests also did not differ significantly from the standard population mean, except for their mean score on Spelling, which fell below the standard mean. There were no significant differences between the mean scores of the ECMO and CM subgroups on any of the tests of academic attainments.

#### Verbal Fluency

Similar results were obtained on the fluency measures. Here, neither measure revealed impairment in the full AHRF cohort. Interestingly, however, on Category Fluency, the patient cohort performed significantly above the standard population mean (*P* = 0.003), due to the attainment of a high mean score in the CM subgroup, selectively (CM vs. ECMO in the full cohort, *P* = 0.02).

#### Memory

Despite preservation of their general cognitive status, the children in the patient cohort were markedly impaired relative to the standard population mean of 100 ± 15 (SD) on nearly every one of the CMS measures. The only exceptions were the subdomain of Visual Immediate Recall and the nonmnemonic measure of Attention/Concentration, on both of which they scored in the average range. In addition, the mean MQ of the full cohort was significantly below the MQ predicted by their Full Scale IQ (mean predicted MQ = 99, SD = 7; mean actual MQ = 87, SD = 17; *t*_(39)_ = −5.55, *P* < 0.001). The patients also showed a marked deficit on the RBMT (*P* = 0.001, Mann–Whitney *U*-test) relative to the score of the normal control group.

Among the full cohort, 18 patients obtained MQ scores significantly below those predicted by their Full Scale IQs (mean = 94, SD = 11; mean predicted MQ = 96, SD = 7; mean actual MQ = 74, SD = 11; *t*_(17)_ = −13.7, *P* < 0.001.

The only memory measures on which the ECMO and CM subgroups differed consistently, that is, within both the full cohort and the randomized trial cohort, were on Learning and Delayed Recognition, in both of which the ECMO subgroup scored below the CM subgroup (Learning: *P* = 0.04 and 0.02 for the full cohort and the randomized trial cohort, respectively; Delayed Recognition: *P* = 0.05 and 0.06 for the full cohort and the randomized trial cohort, respectively).

### Factor Structure of the Cognitive Profile

Substantial correlations were observed between memory measures and measures of intelligence and academic attainments. To determine whether each of these 3 domains contributed to an independent factor, we performed a PCA on the standardized scores of WISC, WIAT, and CMS indices and obtained 3 components. The Kaiser–Meyer–Olkin measure of sampling adequacy for the PCAs was 0.81, and Bartlett's test of sphericity was significant (*χ***²**_(120)_ = 383.42, *P* < 0.001). The 3 extracted components, accounting for 69% of the original variance, were composed of the following measures (with only those component loadings that were > 0.6 reported here): The first component (eigenvalue, 7.06; variance, 44%), subsequently referred to as “Memory” was composed of CMS subtests Verbal Immediate Memory, Verbal Delayed Memory, Visual Immediate Memory, Visual Delayed Memory, Delayed Recognition, and Learning. The second component (eigenvalue, 2.16; variance, 14%) subsequently labeled “Verbal Intelligence,” included WIAT subtests Word Reading, Reading Comprehension, Spelling, Mathematical Reasoning, and the WISC subtest Verbal Comprehension. The third component (eigenvalue, 1.75; variance, 11%) labeled “Attention” included CMS subtest Attention/Concentration and the WISC subtest Working Memory. Regression-based component scores (*M* = 0, SD = 1) were extracted for each participant, and correlations were run between these component scores and left, right, and bilateral HVs.

### Correlations Between Brain Volumes and Neuropsychological Measures

**Figure 2.**
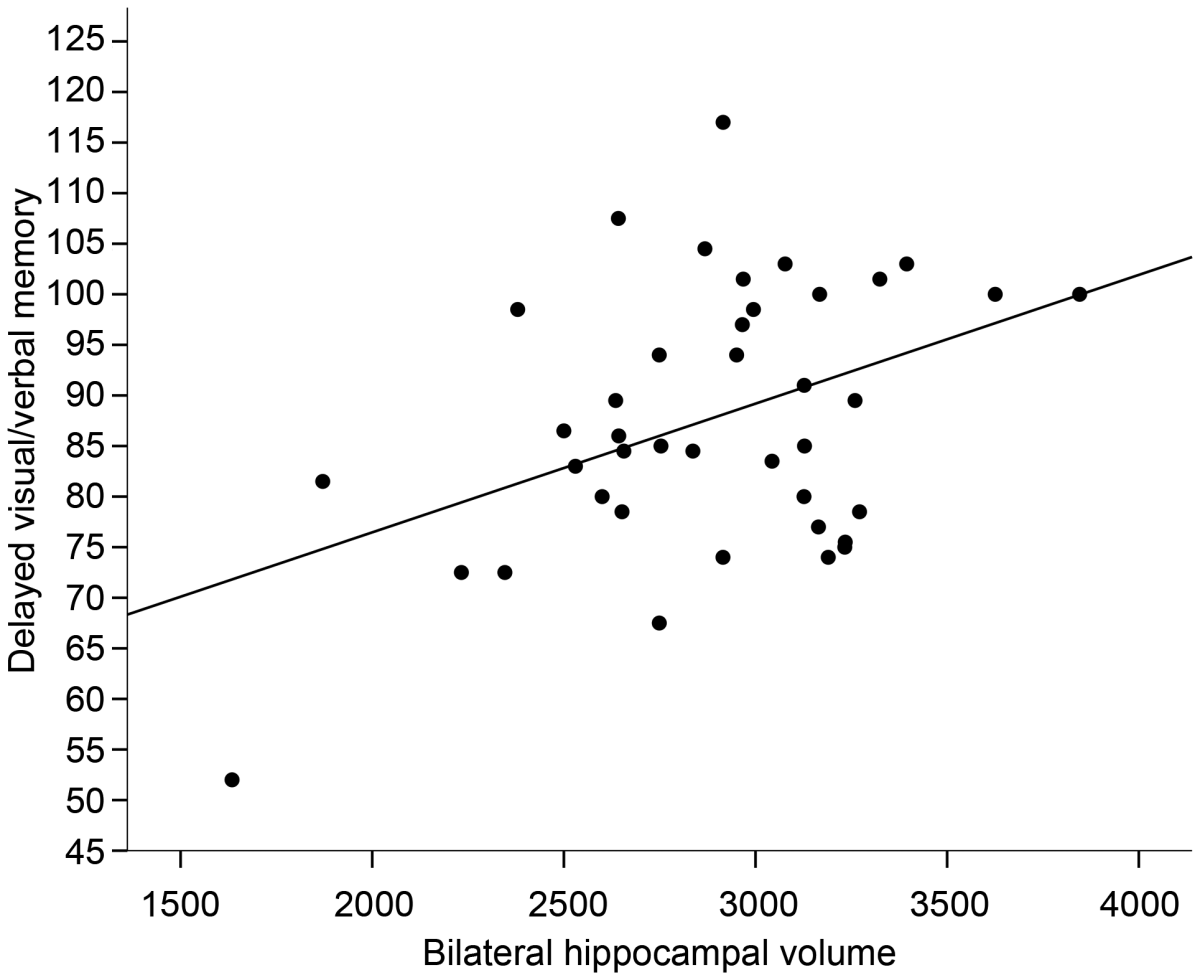
Correlation between bilateral hippocampal volume and mean score on Delayed Visual/Verbal Memory in the AHRF cohort.

In the AHRF cohort, significant positive correlations were found between left, right, and bilateral HVs and both the General Memory Quotient and Delayed Visual/Verbal Memory (see Table 4 and Fig. 2). Among the 3 PCA components (see above), the only one that correlated with HVs was the Memory component (left, *r* = 0.45, *P* = 0.003; right, *r* = 0.34, *P* = 0.031; and bilateral, *r* = 0.41, *P* = 0.009).

**Table 4:** Correlations between hippocampal volumes and measures of memory and learning

	Left hippocampal volume	Right hippocampal volume	Bilateral hippocampal volume
CMS General Memory	*r* = 0.410 *P* = 0.009	*r* = 0.325 *P* = 0.041	*r* = 0.381 *P* = 0.015
CMS Mean Visual/Verbal Immediate Memory	*r* = 0.409 *P* = 0.009	NS^a^	*r* = 0.372 *P* = 0.018
CMS Mean Visual/Verbal Delayed Memory	*r* = 0.437 *P* = 0.005	*r* = 0.375 *P* = 0.017	*r* = 0.421 *P* = 0.007
CMS Learning	*r* = 0.331 *P* = 0.037	NS	NS
CMS Attention/Concentration	NS	*r* = −0.331 *P* = 0.037	NS
Memory Component (PCA)	*r* = 0.451 *P* = 0.003	*r* = 0.341 *P* = 0.031	*r* = 0.410 *P* = 0.009

^a^NS, no significant correlation.

In addition, there were significant positive correlations between left and bilateral HVs and Immediate Visual/Verbal Memory, and between left HV and Learning, but there was no significant correlation between HVs and Full Scale IQ (*r* = 0.083, *P* = 0.612). The only other significant finding was a negative correlation between right HV and the Attention/Concentration score (*r* = −0.331, *P* = 0.037).

Stepwise linear regression analyses were then performed using left, right, and bilateral HVs as predictor variables of the memory scores. These analyses revealed that only left HV was a significant predictor in the total group and that it accounted for 17% of the variance in the General Memory Quotient (*F*_1,39_ = 7.68, *P* = 0.009; *β* = 0.41), 19% of the variance in mean Delayed Visual/Verbal Memory (*F*_1,39_ = 8.97, *P* = 0.005; *β* = 0.44), and 17% of the variance in mean Immediate Visual Memory (*F*_1,39_ = 7.73, *P* = 0.008; *β* = 0.41).

Four children in the patient cohort had both a neuropathology and neuropsychology profile characteristic of patients with DA (Vargha-Khadem et al. 1997; Gadian et al. 2000). Specifically, despite having Intelligence Quotients in the low average range (84–89), these 4 cases had MQs 22–50 points (mean, 35 points) below the standard mean of 100, and Rivermead scores 5–13 points (mean, 10 points) below the mean control score of 20. The severe mnemonic deficits in these 4 children were associated with HVs that were reduced bilaterally 28–50% (mean, 38%) below the mean HVs of the control participants.

### Relationship of Medical Variables to Hippocampal Volumes and Memory Measures

Stepwise linear regressions using all the medical variables listed in Table 1 revealed that the only significant predictor of HVs in the patient cohort was gestational age: left HV *F*_1,39_ = 8.34, *P* = 0.006, *β* = 0.42; right HV *F*_1,39_ = 4.27, *P* = 0.046, *β* = 0.32; bilateral HV *F*_1,39_ = 6.58, *P* = 0.014, *β* = 0.38. This age factor accounted for 18, 10, and 15% of the variance in left, right, and bilateral HV, respectively, with greater gestational age predicting larger HVs.

None of the medical variables directly predicted any of the memory measures in the patient cohort (see Table 1).

## Discussion

Our results support the prediction that neonatal hypoxia can lead to hippocampal damage and, later in childhood, to an impairment in cognitive memory. The findings thus demonstrate that the 3 events do indeed form a causal sequence. Furthermore, the risk of the final event is substantial: Of the 40 patients in the cohort, 19 (∼48%) had significantly reduced MQs relative to values predicted by their Full Scale IQs. Moreover, 4 patients (10%) met both our anatomical and our mnemonic criteria for DA. Equally important, however, many of the remaining patients also had smaller HVs than the controls, and this too led to a significant degree of memory impairment. As a result, our patient cohort, aged 8–15, had a mean HV and a mean memory score that were substantially below those of the normal controls. Importantly, the cognitive impairment was selective to the domain of memory, as indicated by their achievement of average-range scores on tests of intelligence, academic attainments, and verbal fluency. Moreover, because these findings demonstrate that, over the course of their postnatal development, even those children with significant memory problems (see below) were nevertheless able to acquire normal levels of factual, or semantic, knowledge, their mnemonic difficulties appear to be limited to event, or episodic, memory, further implicating hippocampal damage as the cause of their difficulties (Dikova et al. 1993; Mishkin et al. 1997; Vargha-Khadem et al. 1997, 2003; Gadian et al. 2000; Isaacs et al. 2003; Mañeru et al. 2003; de Haan, Mishkin et al. 2006a; de Haan, Wyatt et al. 2006b; Raman et al. 2006; Titus et al. 2007; Huang et al. 2009; Rodricks et al. 2010).

Evidence of a relationship between memory impairment and hippocampal damage was demonstrated by significant correlations between extent of hippocampal atrophy and memory scores across the entire cohort, and regression analyses indicating that HVs were a significant predictor of memory scores. The observations that memory, verbal intelligence, and attention loaded on different factors, supports the conclusion that hippocampal function is selectively related to memory.

Notably, 4 of the children (10% of the cohort) had the neuropathological and the neuropsychological profile of DA. This degree of memory impairment is known to seriously interfere with carrying out the ordinary tasks of daily life at home and at school, and, later in life, with finding gainful employment and living independently (Vargha-Khadem et al. 1997; Gadian et al. 2000; Isaacs et al. 2003).

Despite the chronic impairment in memory after neonatally sustained bilateral hippocampal damage, plastic changes and compensatory mechanisms may be present in the developing young brain. For example, the negative correlation between right HV and the Attention/Concentration score might represent one such compensatory mechanism. Thus, in the presence of a small right hippocampus, extra attentional and concentration efforts may have had to be expended to compensate for the compromised neural substrate. Such compensatory phenomena have been reported in fMRI studies of groups with neurodevelopmental disorders, such as dyscalculia and phonological impairment (Eden et al. 2004; Kaufman et al. 2009).

Having determined that there is a relationship between amount of hippocampal atrophy and degree of memory impairment, it is important to point out the deviations from this relationship. First, although the ECMO and CM subgroups did not differ in degree of hippocampal atrophy, the ECMO subgroup was impaired relative to the CM subgroup in Learning, Delayed Recognition, and Verbal Comprehension (findings observed within both the full cohort and the randomized trial cohort); given the absence of greater hippocampal atrophy in the ECMO subgroup, the only observed pathology that could conceivably account for their memory and comprehension deficits is this subgroup's increased CSF and trend toward reduced global WM. Second, substantial hippocampal damage is not an obligatory consequence of AHRF; many of the children in the cohort had HVs that matched or exceeded the mean HV of the control group (3254 ± 250 mm^3^). In some cases, this may have been a consequence of gestational age: as indicated by the regression models for the effects of the medical variables, the size of the hippocampi, particularly on the left, was predicted by gestational age, suggesting that the closer the newborns had been to normal gestational age, the less vulnerable they were to hippocampal atrophy. A possibility worth considering is that other patients who did not succumb to the adverse consequences of neonatal hypoxia may have had a genetic protection against hypoxia-induced brain damage (Jeong and Park 2012; Lopez-Hernández et al. 2012).

Granting these potential forms of protection, the present findings add to the growing body of evidence that the hippocampus is highly and selectively susceptible to injury caused by hypoxia sustained early in life and that, because the episodic memory functions of the hippocampus do not play a critical role in infancy or even in early childhood, the impairment can remain silent and unnoticed for several years. Acute neonatal respiratory failure is only one of several neonatal events that can lead to hippocampal damage, and, as the present findings show, neonates who suffer such an episode can grow into an unheralded memory impairment that will interfere, and in some cases seriously interfere, with the rest of their lives.

To our knowledge, this is the first demonstration of a predicted relationship between degree of hippocampal damage and degree of memory impairment in a large cohort of children at risk for these effects due to neonatal hypoxia. Wider recognition of this hypoxia-induced neuropathology and memory loss may lead, through combined basic and clinical research, to the development of brain protective measures that will help prevent this adverse causal sequence.

## Supplementary Material

Supplementary material can be found at: http://www.cercor.oxfordjournals.org/

## Funding

This work was supported by the Medical Research Council (program grant number G03000117/65439); the Central and East London Research Network (5177); and the Intramural Research Program of the National Institute of Mental Health, National Institutes of Health, Department of Health and Human Services. Funding to pay the Open Access publication charges for this article was provided by Research Councils UK.

## Supplementary Material

Supplementary Data
