# Supplementary material for: Neonatal Hypoxia, Hippocampal Atrophy, and Memory Impairment: Evidence of a Causal Sequence
Source: Cereb Cortex. 2013 Dec 15;25(6):1469–76. doi: 10.1093/cercor/bht332 (PMC4428295; doi:10.1093/cercor/bht332)
Supplement: Supplementary Data [file supp_25_6_1469__index.html]

Neonatal Hypoxia, Hippocampal Atrophy, and Memory Impairment: Evidence of a Causal Sequence — Neonatal Hypoxia, Hippocampal Atrophy, and Memory Impairment: Evidence of a Causal Sequence — Supplementary Data 

# Neonatal Hypoxia, Hippocampal Atrophy, and Memory Impairment: Evidence of a Causal Sequence

## Supplementary Data

Supplementary Data

**Files in this Data Supplement:**

- Supplementary Data - Pdf file
