# Supplementary material for: Neonatal Hypoxia, Hippocampal Atrophy, and Memory Impairment: Evidence of a Causal Sequence
Source: Cereb Cortex. 2013 Dec 15;25(6):1469–76. doi: 10.1093/cercor/bht332 (PMC4428295; doi:10.1093/cercor/bht332)
Supplement: Supplementary Data [file supp_bht332_bht332supp.pdf]

Supplementary Table 1 – Aetiology in the AHRF cohort

Primary Diagnosis	**N**			**% of cohort**	
Primary Pulmonary Hypertension of the Newborn as a result of:					
Meconium Aspiration Syndrome	19			47.5	
Sepsis	3			7.5	
Respiratory Distress Syndrome	6			15	
Other	5			12.5	
Isolated Persistent Fetal Circulation	4			10	
Hypoplastic Lungs	1			2.5	
Congenital Diaphragmatic Hernia	1			2.5	
Treatment					
ECMO	27			67.5	
CM	13			32.5	
ECMO patients		**Mean**	**SD**	**Median**	**IQR**
Time on ECMO (mins)	27	111.3	37	112	82-125
ECMO Cannulation				**% of ECMO patients**	
Venoarterial [S1]	21			78%	
Venovenous [S2]	6			22%	

*1 patient did not have any information regarding clinical diagnosis

[S1] Venoarterial: Blood is drained from the venous system and returned to the arterial system. This configuration provides both cardiac and respiratory support.

[S2] Venovenous: Blood is drained from the venous system and returned to venous system only provides respiratory support.

Supplementary Table 2A: Medical Variables for the full ECMO and CM subgroups

**Medical Variables**	**ECMO (N=27)**		**CM (N=13)**		**T-test**	
	Mean	SD	Mean	SD	t	p
Gestational age (weeks)	39.4	2.2	39.7	1.9	-0.47	0.64
Birth Weight (kg)	3.5	0.8	3.5	0.6	0.20	0.84
Arterial pH value	7.3	0.1	7.2	0.1	2.33	**0.03**
Arterial CO_2 level (Kpa -before treatment)	5.6	1.9	6.8	2.0	-1.73	0.09
Arterial PaO_2 level (Kpa -before treatment)	5.3	1.6	4.8	1.8	0.80	0.43
Oxygenation Index	59.4	21.4	67.6	23.1	-1.05	0.30

Supplementary Table 2B: Medical Variables for the randomized trial ECMO and CM subgroups

**Medical Variables**	**ECMO (N=17)**		**CM (N=11)**		**T-test**	
	Mean	SD	Mean	SD	t	p
Gestational age (weeks)	39.2	2.1	39.7	2.2	-0.52	0.61
Birth Weight (Kg)	3.7	0.9	3.5	0.6	0.49	0.63
Arterial pH value	7.4	0.1	7.3	0.1	2.31	**0.03**
Arterial CO_2 level (Kpa-before treatment)	5.1	1.8	6.7	2.0	-2.05	**0.05**
Arterial PaO_2 level (Kpa-before treatment)	5.2	1.4	4.5	1.5	1.25	0.22
Oxygenation Index	57.9	23.0	71.4	20.5	-1.54	0.14

Abbreviations: CO_2, carbon dioxide level in arterial blood; kg, kilograms; kPa, kilopascals; PaO_2, partial pressure of oxygen; pH, the acidity/alkalinity of arterial blood; SD, standard deviation.

Supplementary Table 3: SES and CBCL in the full ECMO and CM cohorts

	ECMO				CM				T-Test ECMO vs. CM		
**A. Socio-Economic Status**	**N**	**Mean**	**SD**	**Range**	**N**	**Mean**	**SD**	**Range**	**df**	**t**	**p**
Father's occupation	22	49.9	22.6	23-88	12	48.6	19.9	23-87	32	0.17	0.87
Mother's occupation	20	48.2	18.3	23-88	9	52.2	17.8	25-85	27	-0.56	0.58
**B. Child Behaviour Checklist (CBC)**	**N**	**Mean**	**SD**	**Range**	**N**	**Mean**	**SD**	**Range**	**df**	**t**	**p**
Anxious-Depressed	24	54.4	7.2	50-79	12	53.8	7.2	50-74	34	0.25	0.81
Withdrawn-Depressed	24	55.5	6.7	50-73	12	53.3	4.0	50-60	34	1.07	0.29
Somatic complaints	24	56.4	7.6	50-78	12	55.9	6.2	50-67	34	0.20	0.85
Social problems	24	56.7	8.0	50-70	12	52.7	5.3	50-64	31	1.78	0.08
Thought problems	24	56.9	8.2	50-73	12	53.3	4.6	50-63	33	1.70	0.10
Attention problems	24	58.9	10.1	50-83	12	55.8	7.9	50-73	34	0.92	0.36
Delinquent Behaviour (Rule Breaking)	24	52.9	5.0	50-71	12	51.8	2.4	50-57	34	0.76	0.45
Aggressive Behaviour	24	53.5	7.1	50-75	12	53.2	3.9	50-59	34	0.15	0.88

Supplementary Table 4: SES and CBCL in the ECMO and CM Randomized-trial cohorts

	ECMO				CM				T-Test ECMO vs. CM		
**A. Socio-Economic Status**	**N**	**Mean**	**SD**	**Range**	**N**	**Mean**	**SD**	**Range**	**df**	**t**	**p**
Father's occupation	13	42.0	16.8	23-68	10	49.2	19.2	26-87	21	-0.96	0.35
Mother's occupation	12	44.3	16.3	23-77	8	55.6	15.6	38-85	18	-1.54	0.14
**B. Child Behaviour Checklist (CBC)**	**N**	**Mean**	**SD**	**Range**	**N**	**Mean**	**SD**	**Range**	**df**	**t**	**p**
Anxious-Depressed	15	56.3	8.5	50-79	10	53.2	7.4	50-74	23	0.93	0.37
Withdrawn-Depressed	15	56.7	6.8	50-73	10	53.1	3.9	50-60	23	1.50	0.15
Somatic complaints	15	58.0	9.0	50-78	10	56.4	6.5	50-67	23	0.49	0.63
Social problems	15	56.5	8.3	50-70	10	53.2	5.7	50-64	23	1.09	0.29
Thought problems	15	57.7	8.7	50-73	10	53.8	4.8	50-63	22	1.45	0.16
Attention problems	15	58.8	10.2	50-83	10	56.7	8.4	50-73	23	0.54	0.59
Delinquent Behaviour (Rule Breaking)	15	54.1	6.0	50-71	10	52.0	2.6	50-57	23	1.02	0.32
Aggressive Behaviour	15	54.5	8.5	50-75	10	53.8	4.0	50-59	23	0.23	0.82

Supplementary Table 5: Brain volumes of the full AHRF cohort and the randomized-trial cohort.

	Full AHRF Cohort							*Randomized-trial cohort*					
	**ECMO (N=27)**		**CM (N=13)**		**T-Test ECMO vs CM**			**ECMO (N=17)**		**CM (N=11)**		**T-Test ECMO vs CM**	
Region	***Mean (SS)***	***SD***	***Mean (SS)***	***SD***	***t (df=38)***	***p***	***Region***	***Mean (SS)***	***SD***	***Mean (SS)***	***SD***	***t (df=38)***	***p***
**Hippocampal Volumes (mm^3)**							**Hippocampal Volumes (mm^3)**						
Left	2782	469	2991	321	-1.44	0.16	Left	2830	510	3080	252	-1.5	0.15
Right	2821	498	3096	319	-1.82	0.08	Right	2883	585	3133	335	-1.28	0.21
Mean	2801	469	3043	290	-1.7	0.10	Mean	2856	533	3106	268	-1.44	0.16
**Other brain volumes (cm^3)**							**Other brain volumes (cm^3)**						
Grey Matter	738	14	741	13	-0.50	0.62	Grey Matter	733	14	739	14	-1.28	0.21
White Matter	494	14	501	11	-1.72	0.09	White Matter	496	16	501	13	-0.91	0.37
CSF	283	15	273	12	2.14	**0.04**	CSF	286	16	274	12	2.17	**0.04**

Supplementary Table 6: Cognitive profiles of the full AHRF cohort and the randomized-trial cohort

Full AHRF Cohort

Cognitive Domain	*ECMO (N=27)*		*CM (N=13)*		*T-Test ECMO vs CM*	
	Mean (SS)	***SD***	***Mean (SS)***	***SD***	***t (df=38)***	***p***
**Intelligence (WISC-IV)**						
Verbal Comprehension	94	12	102	12	-2.25	**0.03**
Perceptual Reasoning	98	14	103	15	-1.04	0.30
Working Memory	98	12	92	10	1.59	0.12
Processing Speed	97	14	104	13	-1.55	0.13
Full Scale IQ	95	12	101	13	-1.42	0.16
**Academic Attainments (WIAT)**						
Word Reading	98	13	97	9	0.21	0.84
Reading Comprehension	101	15	104	13	-0.67	0.51
Spelling	94	14	97	7	-0.88	0.39
Numerical Operations	97	20	91	13	0.94	0.36
Mathematical Reasoning	98	12	97	11	0.31	0.76

Randomized-trial cohort

Cognitive Domain	*ECMO (N=17)*		*CM (N=11)*		*T-Test ECMO vs CM*	
	Mean (SS)	***SD***	***Mean (SS)***	***SD***	***t (df=26)***	***p***
**Intelligence (WISC-IV)**						
Verbal Comprehension	92	9	101	12	-2.01	**0.05**
Perceptual Reasoning	96	14	101	15	-0.91	0.37
Working Memory	96	9	89	7	2.12	**0.04**
Processing Speed	95	10	102	13	-1.52	0.14
Full Scale IQ	93	9	99	12	-1.35	0.19
**Academic Attainments (WIAT)**						
Word Reading	99	12	94	6	1.23	0.23
Reading Comprehension	100	15	102	13	-0.33	0.75
Spelling	93	11	96	6	-1.03	0.31
Numerical Operations	95	19	90	14	0.72	0.48
Mathematical Reasoning	99	10	95	11	1.06	0.30

**Verbal Fluency (D-KEFS)**							**Verbal Fluency (D-KEFS)**						
Letter Fluency	98	18	99	14	-0.89	0.93	Letter Fluency	99	15	96	14	0.39	0.7
Category Fluency	104	12	114	16	-2.38	**0.02**	Category Fluency	102	13	112	16	-1.73	0.1
**Memory (CMS)**							**Memory (CMS)**						
General Memory Quotient	84	19	93	10	-2.06	**0.05**	General Memory Quotient	83	19	92	10	-1.36	0.19
Verbal Immediate	83	16	92	10	-1.92	0.06	Verbal Immediate	83	16	91	10	-1.51	0.14
Verbal Delayed	82	16	92	12	-1.91	0.06	Verbal Delayed	83	17	90	11	-1.2	0.24
Visual Immediate	99	14	101	10	-0.36	0.72	Visual Immediate	99	14	102	10	-0.62	0.54
Visual Delayed	88	15	93	11	-1.19	0.24	Visual Delayed	87	16	93	12	-1.10	0.28
Attention/Concentration	104	15	98	11	1.24	0.22	Attention/Concentration	101	15	96	11	0.99	0.33
Learning	90	13	99	12	-2.1	**0.04**	Learning	89	12	101	12	-2.59	**0.02**
Delayed Recognition	80	17	92	20	-2.01	**0.05**	Delayed Recognition	76	16	90	21	-1.95	**0.06**
Mean of Visual/Verbal Immediate	91	13	97	8	-1.34	0.19	Mean of Visual/Verbal Immediate	91	13	96	9	-1.26	0.22
Mean of Visual/Verbal Delayed	85	14	93	10	-1.78	0.08	Mean of Visual/Verbal Delayed	85	15	91	9	-1.28	0.21
**Rivermead Behavioural Memory Test (/22) (Mann-Whitney U)**	m*	SD	m	SD	*z*		**Rivermead Behavioural Memory Test (/22) (Mann-Whitney U)**	m	SD	m	SD	*z*	
	16	5	18	3	-1.3	0.2		16	5	18	2	*-0.66*	0.52

* m= median
